# Identification of bacterial biofilm and the *Staphylococcus aureus* derived protease, staphopain, on the skin surface of patients with atopic dermatitis

**DOI:** 10.1038/s41598-017-08046-2

**Published:** 2017-08-18

**Authors:** Andreas Sonesson, Kornelia Przybyszewska, Sigrid Eriksson, Matthias Mörgelin, Sven Kjellström, Julia Davies, Jan Potempa, Artur Schmidtchen

**Affiliations:** 1 Division of Dermatology and Venereology, Department of Clinical Sciences Lund, Lund University, BMC, Tornavägen 10, SE-22184 Lund, Sweden; 2 Dermatology and Venereology, Skane University Hospital, Lasarettsgatan 15, SE-22185 Lund, Sweden; 3 Department of Microbiology, Faculty of Biochemistry, Biophysics and Biotechnology, Jagiellonian University, 30-387 Krakow, Poland; 4 Division of Infection Medicine, Department of Clinical Sciences, Lund University, Biomedical Center B14, 221 84 Lund, Sweden; 5 Department of Biochemistry and Structural Biology, Center for Molecular Protein Science, Lund University, PO Box 124, Lund, SE-22362 Sweden; 6 Department of Oral Biology, Faculty of Odontology, Malmö University, 20506 Malmö, Sweden; 7 Malopolska Center of Biotechnology, Jagiellonian University, 30-387 Krakow, Poland; 8 Department of Oral Immunology and Infectious Diseases, University of Louisville School of Dentistry, Louisville, KY 40202 USA; 9 Dermatology, LKCMedicine, Nanyang Technological University, Singapore, 636921 Singapore; 10 Center for Infection and Immunity, School of Medicine, Dentistry and Bio-medical Sciences Queen’s University Belfast, Belfast, UK

## Abstract

Atopic dermatitis (AD) is a chronic inflammatory skin disease characterized by an impaired epidermal barrier, dysregulation of innate and adaptive immunity, and a high susceptibility to bacterial colonization and infection. In the present study, bacterial biofilm was visualized by electron microscopy at the surface of AD skin. Correspondingly, *Staphylococcus aureus* (*S*. *aureus*) isolates from lesional skin of patients with AD, produced a substantial amount of biofilm *in vitro*. *S*. *aureus* biofilms showed less susceptibility to killing by the antimicrobial peptide LL-37 when compared with results obtained using planktonic cells. Confocal microscopy analysis showed that LL-37 binds to the *S*. *aureus* biofilms. Immuno-gold staining of *S*. *aureus* biofilm of AD skin detected the *S*. *aureus* derived protease staphopain adjacent to the bacteria. *In vitro*, staphopain B degraded LL-37 into shorter peptide fragments. Further, LL-37 significantly inhibited *S*. *aureus* biofilm formation, but no such effects were observed for the degradation products. The data presented here provide novel information on staphopains present in *S*. *aureus* biofilms *in vivo*, and illustrate the complex interplay between biofilm and LL-37 in skin of AD patients, possibly leading to a disturbed host defense, which facilitates bacterial persistence.

## Introduction

Atopic dermatitis (AD) is a chronic inflammatory skin disease usually starting in early childhood with a reported lifetime prevalence of 15–30% in children, whereas the corresponding figure for adults is 2–10%^1, 2^. The pathogenesis of AD involves genetic factors as well as gene-environmental interactions. Several genes associated with skin barrier dysfunction, proteolytic activity, and the immune system are found to be associated with AD, and play a role in the etiology of the dysfunctional skin barrier and the immunological abnormalities found in AD skin. The disease is characterized by a relapsing course and the clinical presentations of AD are severe pruritus, xerosis, an increased transepidermal water loss, typically distributed eczema lesions in flexural areas, and recurrent cutaneous infections. Skin colonization by *Staphylococcus aureus* (*S*. *aureus*) is prevalent in AD, and seems to be promoted by skin barrier impairment^3, 4^. *S*. *aureus* bacteria play an important role in the pathogenesis of AD by secreting toxins, antigens, and proteases that interact with keratinocytes and various inflammatory cells, and lead to dysregulation of skin hemostasis, which ultimately compromises the epidermal barrier^5^. *In vivo*, biofilms are the predominant bacterial mode of life and may contribute to the pathogenesis of various chronic infections such as periodontitis, orthopedic infections, and non-healing skin ulcers^6^. Moreover, biofilms have also been detected on the skin of AD patients, and may possibly contribute to the inflammatory process^7–9^. Biofilms are bacterial agglomerations attached to a surface and embedded in an extracellular matrix^10^. Formation of biofilm is a successful strategy that protects the bacteria from environmental danger, antimicrobial peptides (AMPs), antibiotics and phagocytosis^11, 12^, enabling chronic persistence in the host. An increasing number of *S*. *aureus* skin isolates are resistant to conventional antibiotics and today, there is an unmet need for novel strategies to effectively counteract skin infections.

Resident cells of the epidermis, such as the keratinocytes have evolved mechanisms to combat colonization by pathogenic bacteria. One crucial strategy utilized by the epidermal cells is to secrete AMPs^13^. AMPs constitute a part of the innate immune defense and are of fundamental importance, in particular in relation to the often observed bacterial colonization of AD skin lesions^14^. In general AMPs are small cationic peptides with hydrophobic properties that interact with the target microorganism, preferentially negatively charged structures of the bacterial membrane, and form pores that eventually causes lysis of the cell^15^. In human skin several AMPs have been identified and are considered essential effector molecules of innate immunity^16^. In some cases, truncated parts of AMPs are detected during skin inflammation. For example, fragments of the cathelicidin peptide LL-37, generated by cutaneous protease activity, have been described in skin diseases such as acne rosacea^17, 18^. Furthermore, *S*. *aureus* derived proteinases, such as aureolysin and V8 protease, have previously been shown to degrade the human AMP LL-37^19^. Along with aureolysin and V8, the cysteine proteases staphopain A (ScpA) and B (SspB) are additional major proteases secreted by *S*. *aureus*. SspB is known to contribute to bacterial virulence, whereas ScpA seems to have another unrelated function^20^. Interestingly, the presence of V8 protease and aureolysin has been observed in staphylococcal isolates from AD patients^21^. However, currently there is no information regarding the expression of staphopains in AD isolates.

The aim of this study was to investigate the *in vitro* biofilm production by *S*. *aureus* isolates, derived from skin lesions of AD patients. Furthermore we aimed to investigate if bacterial proteases, such as staphopains, are present at the skin surface of these patients. Moreover, we wanted to study, *in vitro*, if these proteases degraded LL-37, an important innate defense molecule found in inflamed skin.

## Results

### Visualization of bacterial biofilm in lesional skin of patients with AD

**Figure 1:**
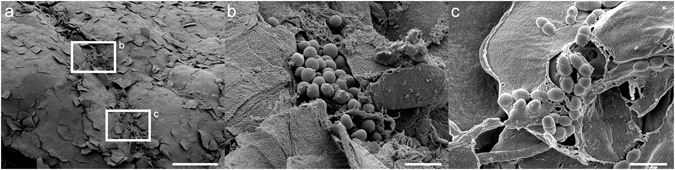
The presence of bacterial biofilm at lesional AD skin colonized with *S*. *aureus*. Biofilm observed by scanning electron microscopy on the stratum corneum of an AD patient. (**a**) Microbial biofilm on skin of an AD patient colonized with *S*. *aureus*. The high magnification pictures in figures b and c are derived from the area in box **b** and **c**. Scale bar 100 μm. (**b**) Biofilm composed of intricate extracellular matrix material and bacteria. Scale bar 10 μm. (**c**) High magnification view visualizing individual bacteria surrounded by extracellular matrix material observed between corneocytes on AD skin. Scale bar 2 μm.

Scanning electron microscopy (SEM) analysis of skin biopsies from *S*. *aureus* colonized lesional skin of AD patients demonstrated the presence of bacteria and bacterial biofilm at the skin surface. The results showed biofilm at structures identified in the stratum corneum composed of corneocytes, intricate extracellular matrix material, and bacteria. Moreover, it was also possible to visualize individual bacteria surrounded by extracellular matrix material between the corneocytes (Fig. 1a–c). When biopsies of non-lesional skin derived from a non-infected AD patient (Supplementary Fig. S1a,b) and from lesional skin of three AD patients (with *S*. *aureus* verified skin colonization) (Supplementary Fig. S1c–h) were investigated using SEM, the results showed that lesional AD skin contained considerably more fibrin, extracellular material, and bacteria when compared to non-lesional skin. Next, an *ex vivo* model was used to quantify the presence of strongly adherent bacteria, representing bacterial biofilm in skin biopsies from patients with AD. The quantification of adherent bacteria was done by enumerating colony-forming units (CFU) per square centimetre of skin released after the biopsies were washed, vortexed and sonicated. The results showed that both weakly (median value 1.01 × 10^4^ CFU/cm^2^) and strongly (median value 1.38 × 10^4^ CFU/cm^2^) attached bacteria were present in the AD skin samples (Supplementary Fig. S1i).

### Biofilm production by *S*. *aureus* isolates derived from skin of patients with AD

Biofilm formation was measured among 32 isolates of *S*. *aureus* derived from skin of AD patients and molecular typing was performed using ADSRRS-fingerprinting analysis. The results showed that 12 out of 32 of the isolates (38%) have the ability to produce a substantial amount of biofilm *in vitro*, as detected using the crystal violet method (Supplementary Table S1 and Supplementary Fig. S2). According to the molecular typing data, the capacity to produce biofilm was not associated with any specific strain among the skin-derived *S*. *aureus* isolates, (Supplementary Table S1). Notably, six of the isolates showed strong capacity to produce biofilm *in vitro*, yielding an OD_600_-value in the biofilm assay of more than 0.6 (Supplementary Fig. S2).

It has been recently shown by SEM that the organization of *S*. *aureus* bacteria undergo gradual morphological changes at different growth phases during biofilm formation^22^. To determine if the differences in biofilm formation were dependent on the growth of the bacterial isolates, the optical density of liquid cultures was measured spectrophotometrically. The results showed that growth rates were approximately similar among the tested *S*. *aureus* strains (Supplementary Fig. S3), and no significant differences between strong and weak biofilm-producing strains were observed.

### Antimicrobial effects of LL-37

To test if the human cathelicidin peptide LL-37 exerted different antimicrobial effects on planktonic bacteria and on bacterial biofilm, a modified version of the minimal biofilm eradication concentration (MBEC) assay was used on various biofilm producing *S*. *aureus* isolates, all derived from skin of patients with AD. The results showed a marked difference between the minimum inhibitory concentration (MIC) values and the MBEC values, and indicated that the concentration of LL-37 needed to eradicate bacterial biofilms exceeded the highest tested concentration of 160 μM (Table 1). In contrast, the MIC values obtained for planktonic cells were in the range of 10–20 μM LL-37 (Table 1).

**Table 1:** Measurement of the bacterial susceptibility to the AMP LL-37 was determined by using a modified version of the Calgary Biofilm Device (CBD) method^59^.

Strain ID	Genotype ADSRRS-fingerprinting	Biofilm-Production OD_600nm_ Mean (SD)	LL-37 (μΜ)	
			MIC	MBEC
13	E	0.89 (0.09)	10	>160
5	N	0.16 (0.04)	20	>160
3	S	0.20 (0.06)	10	>160
24	L	1.08 (0.11)	20	>160
ATCC 29213	NA	0.42 (0.09)	20	>160

Minimum Inhibitory Concentration (MIC) and Minimal Biofilm Eradication Concentration (MBEC) are assessed among two low and two high biofilm producing isolates derived from skin of patients with AD, as well as the ATCC strain 29213.

### Visualization by confocal light scanning microscopy (CLSM) of human LL-37 binding to bacterial biofilm

**Figure 2:**
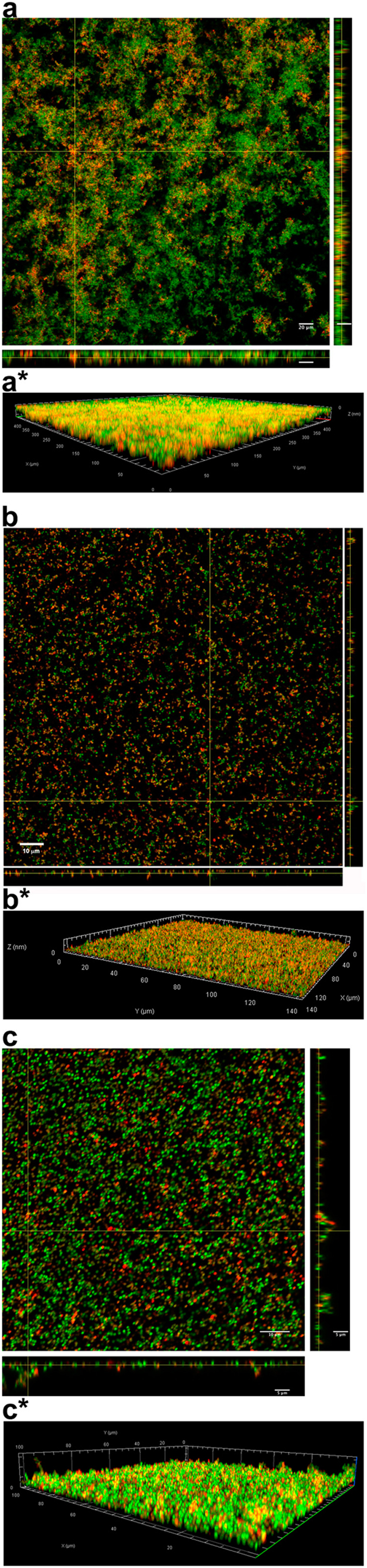
LL-37 binding to bacterial biofilm. Three *S*. *aureus* isolates, derived from three patients with AD, were grown on Cellview^TM^ cell culture dishes for 6 days to form mature biofilms. After washing away the planktonic cells, the bacterial cells of the biofilm (green) were stained with SYTO^®^ 9 green fluorescent stain and then incubated with TAMRA-labeled LL-37 (red), co-existence of bacterial cells and the TAMRA-labeled LL-37 peptide is represented by yellow color. CLSM orthogonal images of Z-stacks show a plane view (square) looking down the biofilm and side views through the biofilm (right and below). Magnification in (**a**) ×20, bar 20 μm, in (**b**) ×63, bar 10 *μ*m and in (**c**) ×63, bars 10 *μ*m and 5 *μ*m. *3D view of the corresponding biofilms.

To determine whether LL-37 binds to the bacterial biofilm, mature (6-day old) biofilms produced by three *S*. *aureus* isolates, derived from lesional skin of three AD patients, were grown on Cellview^TM^ cell culture dishes with glass bottom. The bacterial cells in the biofilm were stained with the fluorescent stain SYTO^®^ 9 green and incubated with TAMRA labeled LL-37. The results showed that the LL-37 peptide bound to the bacterial cells in the biofilms (Fig. 2). When the Z-stacks were analyzed, and quantification of the intensity obtained by *Profile* plug-in (Zeiss ZEN Confocal Software), results indicated three distinctly different populations of fluorophores in the samples; red (TAMRA labeled LL-37) and green (SYTO^®^ 9) seen separately, and yellow representing co-existence of bacterial cells and the TAMRA-labeled LL-37 peptide. Binding of TAMRA labeled LL-37 to the *S*. *aureus* cells was observed throughout the biofilm (Supplementary Fig. S4a–c).

### *S*. *aureus* staphopain and effects on LL-37

**Figure 3:**
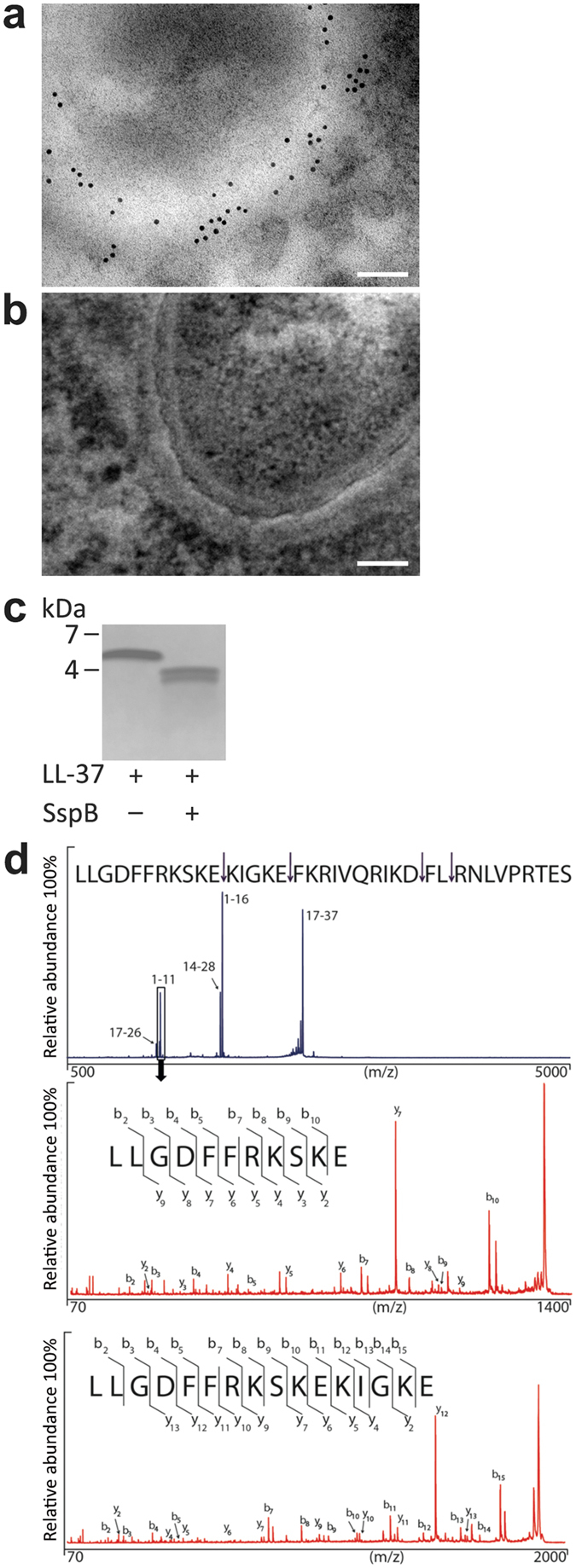
The *S*. *aureus* derived protease staphopain in bacterial biofilm and degradation of LL-37. (**a**) Visualization of staphopain protease at the bacterial surface of cocci-like bacterial structures in skin biopsy from *S*. *aureus* colonized lesional skin of patient with AD. Binding of gold-conjugated antibodies, directed against the *S*. *aureus* derived proteases ScpA and SspB, are shown at the surface of coccoid bacteria. Scale bar 100 nm. (**b**) In the control experiments, no unspecific binding of gold-conjugated secondary IgG antibodies was observed, scale bar 100 nm. (**c**) LL-37 was incubated with and without *S*. *aureus* SspB and analysed using SDS-PAGE (Novex^®^ 10–20% Tricine Gel). Several distinct low molecular weight fragments were observed and further analyzed by MS. (**d**) Mass spectrometry analysis (depicted in blue) of the proteolytic digested LL-37 by SspB, resulting in 100% sequence coverage. The arrows illustrate proteolytic cleavage sites, and MSMS experiment (depicted in red) on the 1–11 peptide resulted in an almost complete y- and b-ion series that identifies the 1–11 sequence LLGDFFRKSKE. Moreover, the analysis revealed an additional fragment (LLG16) (lower panel).

Previous investigations demonstrated that LL-37 could be degraded by the staphylococcal proteases aureolysin and V8 protease^19^. Here, we explored whether the *S*. *aureus* derived protease staphopain was produced by the bacteria *in vivo* and if it could cleave LL-37 *in vitro*. First, biopsies from lesional skin of patients with AD were examined using electron microscopy, with gold-labeled antibodies against the protease. The results showed that the *S*. *aureus* staphopain was indeed present adjacent to coccoid bacteria in the biofilm (Fig. 3a,b). As SspB has been particularly associated with virulence^20^, we first investigated if this protease was able to degrade LL-37. As demonstrated by gel-electrophoresis, LL-37 was degraded into fragments of lower molecular weight by the enzyme. The digested material was analyzed by mass spectrometry (MS) and the results revealed several low molecular weight peptide fragments (Fig. 3c,d). Moreover, when LL-37 was subjected to ScpA the results did not reveal any obvious major cleavage of LL-37 as demonstrated by gel-electrophoresis, although the MS data detected cleavage of minor fragments (Supplementary Fig. S5). These data were thus in correspondence with previous findings of SspB as a major virulence factor.

**Figure 4:**
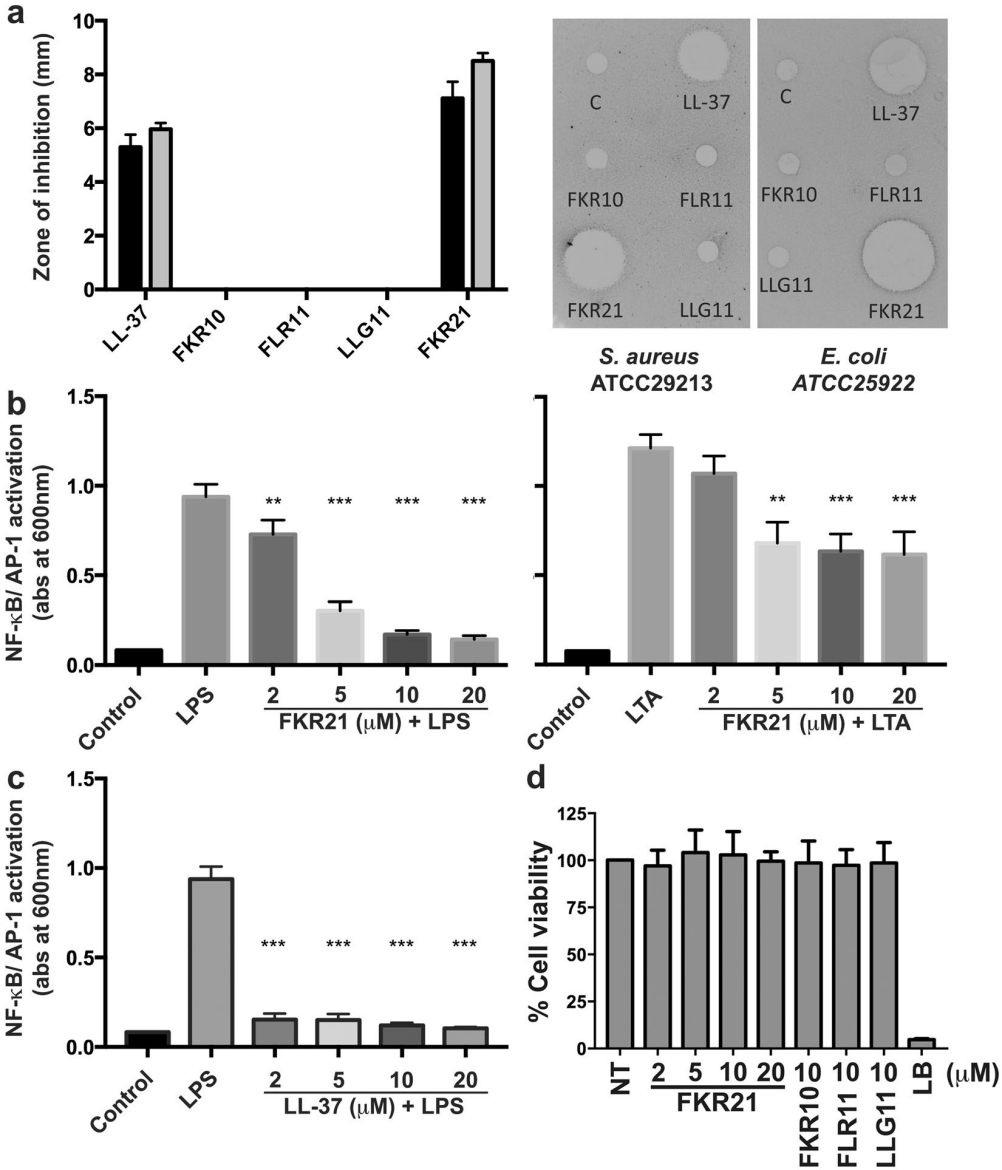
Antimicrobial and immune-modulatory effects of LL-37 and peptide fragments. (**a**) Determination of antimicrobial activity (using radial diffusion assay, RDA) of the peptide fragments (FKR10, FLR11, LLG11 and FKR21) and LL-37 (100 μM) against *S*. *aureus* ATCC29213 (black bars) and *E*. *coli* ATCC25922 (grey bars) (n = 6). The right panel illustrates two scanned examples of representative RDA gels visualizing the zones of clearance corresponding to the inhibitory effects of the peptides against *S*. *aureus* and *E*. *coli* after incubation at 37 °C for 18–24 h (C, control, buffer 10 mM Tris pH 7.4). (**b**) Evaluation of NF-κB/AP-1 activation in supernatants of THP1-X-Blue CD14 cells after stimulation with 100 ng/ml of *E*. *coli* LPS (left) or 1 μg/ml of *S*. *aureus* LTA (right) and increasing concentrations of FKR21. (**c**) Evaluation of NF-κB/AP-1 activation in supernatants of THP1-X-Blue CD14 cells after stimulation with 100 ng/ml of *E*. *coli* LPS and increasing concentrations of LL-37 are shown for comparison. (**d**) Cell viability of HaCaT keratinocytes was analysed using a MTT assay. The results are indicated as mean absorbance values after treatment by the LL-37 derived peptide fragments, which correspond to the amount of living cells, and are compared to the control representing non-treated (NT) cells. Lysis Buffer (LB) yielded 100% lysis of the cells, mean values and SD of at least three independent experiments are presented (**P < 0.01, ***P < 0.001).

To investigate the antibacterial effects of the peptide fragments on planktonic bacteria, a radial diffusion assay was performed. The results demonstrated that only one fragment, FKR21 was antimicrobial against the Gram-positive *S*. *aureus* and Gram-negative *E*. *coli* bacteria (Fig. 4a).

Fragments of LL-37 have previously been ascribed both pro-and anti-inflammatory effects^23–29^. Table 2 compares the identified fragments with previously published LL-37 derived peptide sequences, and references are included illustrating reported immune-modulating actions. To explore if these staphopain-generated peptide fragments also showed immune-modulatory effects *in vitro*, four peptide sequences from the C-terminal part of LL-37 identified by MSMS were synthesized (FKR10, FLR11, LLG11 and FKR21) (Table 2 and Fig. 3d). The peptides were then added to lipopolycaccharide (LPS)- or lipoteichoic acid (LTA)-stimulated human monocytic cells (THP1-XBlue-CD14) to address effects of the fragments on activation of the transcription factors NF-κB and AP-1, central initiators of pro-inflammatory cytokine production^30, 31^. The results showed that that only FKR21 dose-dependently reduced LPS- and LTA-induced NF-κB and AP-1 activation (Fig. 4b), the results of LL-37 are included for comparison (Fig. 4c). To investigate the impact on viability of keratinocytes, by the LL-37 derived peptide fragments, HaCaT cells were subjected to the fragments. The viability was analysed by utilizing the MTT assay. As shown, the HaCaT cells were not significantly affected by the LL-37 derived peptide fragments (Fig. 4d). It is of note that the analysis also revealed an additional minor fragment (LLG16) (Fig. 3d, lower panel), which, interestingly, corresponds to similar fragments previously characterized^23^ and generated by processing of cathelicidin by skin proteases^32^.

**Table 2:** A selection of published peptide sequences derived from LL-37 with immune-modulating actions, and examples of their biological effects.

Name	Sequence	Biological activity	Reference
LL-37	LLGDFFRKSK EKIGKEFKRI VQRIKDFLRN LVPRTES	AM, AB, IM	This paper^23^
LLG11	LLGDFFRKSK E	NF	This paper
LLG16	LLGDFFRKSK EKIGKE	NF	This paper^23^
LLG	LLGDFFRKSK EKIGKEFKRI V	AM, IM^2^	24
GKE	GKEFKRI VQRIKDFLRN LVPR	AM, IM^1c,2^	24
FKR10	FKRI VQRIKD	NF	This paper
FKR21	FKRI VQRIKDFLRN LVPRTES	AM, IM^1a,2^	This paper^24^
FLR11	FLRN LVPRTES	NF	This paper
RK-31	RKSK EKIGKEFKRI VQRIKDFLRN LVPRTES	AM, IM^3^	23, 25
KS-30	KSK EKIGKEFKRI VQRIKDFLRN LVPRTES	AM, IM^3,4^	23, 25, 26
LL-31	LLGDFFRKSK EKIGKEFKRI VQRIKDFLRN L	IM^1b,3^	27
LL-25	LLGDFFRKSK EKIGKEFKRI VQRIK	IM^1b^	27
RK-25	RKSK EKIGKEFKRI VQRIKDFLRN L	IM^1b,3^	27
IG-25	IGKEFKRI VQRIKDFLRN LVPRTES	IM^1b,3^	27
IG-19	IGKEFKRI VQRIKDFLRN L	IM^1b,2,5^	27, 28
F106	GDFFRKSK EKIGKEFKRI VQRIKDFLRN LVPRTES	AM, IM^2^	29
F110	RKSK EKIGKEFKRI VQRIKDFLRN LVPRTES	AM, IM^1c,2^	29

IM, immune-modulatory effects: ^1a^Reduced LPS or LTA induced NF-κΒ and AP-1 activation or ^1b^LPS binding, with implication to neutralize the LPS cytokine response, ^1c^Inhibition of LPS-induced nitric oxide production, ^2^chemotactic activities on human neutrophils, ^3^inhibition of IL-8 production, ^4^increased IFNs production from keratinocytes, ^5^abrogated IL-32γinduced TNF-α and IL-1β production in peripheral blood mononuclear cells (PBMC). AM, antimicrobial effects. AB, anti-biofilm effects. NF, no immune-modulating activity found. F, fragment.

### LL-37 effects on biofilm production

Although we could not find any substantial effect of LL-37 on eradication of biofilm assessed by the Calgary Biofilm Device (CBD)-method, LL-37 might still have inhibitory effects on biofilm formation. To investigate this, an abiotic solid surface assay was performed as described by Hell *et al*.^33^, with minor modifications. The results showed that LL-37 concentrations above 20 μM significantly inhibited biofilm formation of *S*. *aureus* isolates being high producers of biofilm (*P* < 0.05, Wilcoxon rank sum test), whereas no or little inhibitory effect was observed on low biofilm producing isolates, as well as the ATCC29213 strain (Supplementary Fig. S6a). The LL-37 derived fragments yielded no effects on biofilm formation at the same doses as used for LL-37 (Supplementary Fig. S6b). Moreover, *S*. *aureus* isolates were subjected to LL37 and FKR21 in low salt and physiologic salt conditions. The results showed that LL-37 was less inhibited by salt when compared to the results obtained with the peptide FKR21 (Supplementary Fig. S7).

## Discussion

The main findings in this study are the identification of bacterial biofilm in lesions of *S*. *aureus* colonized AD skin combined with findings that *S*. *aureus* isolates of patients with AD are able to produce a substantial amount of biofilm, which in turn, may protect *S*. *aureus* from LL-37-mediated killing. Since biofilms are typical for later stage growth phases, our data correspond well with results showing that protease production of *S*. *aureus* reaches maximal activity in the post-exponential growth phase^20^. Our data also correspond well with recent reports identifying biofilms and glycocalyx structures on skin from AD patients, along with biofilm producing *S*. *aureus* isolates^7, 9, 34^. Taken together, all these observations, combined with the fact that staphopains are among the most copiously produced proteases of *S*. *aureus* ^20, 35^, clearly motivate further studies on the clinical importance of biofilms for bacterial persistence and protease activity in patients with AD.

LL-37 is one of the most well characterized AMPs found in human skin particularly under inflammatory conditions, present in the specific granules of neutrophils and in keratinocytes^36–38^. The level and expression of LL-37 is reported to range from ≈1 μM in human sweat, to higher local concentrations in inflamed skin, such as in acne rosacea^17, 18^. *S*. *aureus* biofilms are composed of, not only bacteria, but also an extracellular matrix comprising multiple macromolecules, including polysaccharides (such as polysaccharide intercellular adhesin), extracellular DNA and proteins^39^. It is therefore likely that interactions between biofilm substances such as negatively charged polysaccharides and LL-37 can lead to scavenging and inactivation of the peptide´s antimicrobial activity. Compatible with these observations are the findings with confocal microscopy, showing binding of LL-37 to the *in vitro* grown biofilm. It is also of note that at high concentrations, LL-37 showed inhibitory effects on biofilm formation of *S*. *aureus*. Hence, our results correspond with earlier reports on LL-37 and biofilm formation, however, the effectiveness of LL-37 in disrupting *S*. *aureus* biofilm seems to be less in comparison to other peptides^40, 41^. Although the exact mechanisms of how LL-37 performs its anti-biofilm effects are not fully elucidated, the central fragment of the peptide seems to be important for its anti-biofilm effects^42^.

The findings that *S*. *aureus* proteases from the biofilm, such as staphopains, were able to degrade LL-37, generating peptide fragments with modified or abrogated antibacterial effects on planktonic bacteria as well as bacterial biofilms, may have important implications. It has been previously reported that peptide fragments produced by degradation of LL-37 can exert pro-inflammatory actions in skin^18^. The degradation of LL-37 by staphopains may therefore yield comparable pro-inflammatory effects which in turn, might further amplify the inflammatory process and the “pathogenic vicious loop” typical for AD^43^. In our *in vitro* studies one of the low molecular weight peptide fragments produced by degradation of LL-37 (FKR21) was able to reduce TLR4- and TLR2-mediated responses, indicating that some fragments may have retained anti-inflammatory actions. Interestingly, a fragment produced by the staphopain SspB (LLG16), is almost identical to a fragment generated by processing of cathelicidin by skin proteases (LL-17)^32^. This suggests a functional overlap between the activities of endogenous proteases such as kallikreins, and bacterial enzymes such as SspB. Furthermore, bacterial proteases found in biofilms can also provide a reservoir of enzymes, that directly activate proenzymes, such as kallikreins, or perhaps cleave structural proteins of importance for maintaining a permeability barrier^44, 45^.

As mentioned in the Introduction, staphylococci may influence various pathomechanisms in AD. The results by Allen *et al*.^7^ indicate that staphylococci may activate toll like receptor 2 (TLR2)^7^ and up-regulate the expression and production of several pro-inflammatory cytokines^14^. Even though it is reported that patients with AD show less response to TLR-2 stimulation than healthy controls^46^, several products from *S*. *aureus* are known to induce a pro-inflammatory response in keratinocytes^47^. Furthermore, superantigens from staphyloccoci may directly stimulate T-lymphocytes via the T-cell receptor^48^. It is also reported that protease production by *S*. *aureus* causes skin barrier dysfunction^49^ and that *S*. *aureus* colonization is associated with impairments of the skin barrier in AD^4^. Chronic biofilm infections by *S*. *aureus* can activate the immune system generating damage of host tissue, and therefore generate an environment facilitating the persistence of the biofilm^50^. Moreover, suppression of the inflammatory response seems to prevent the development of chronic biofilm infections^51^.

Biofilm production by staphylococci could constitute an overlooked pathogenic mechanism, leading to inactivation of AMP-mediated bacterial killing. It is also notable that *S*. *aureus* derived proteases, such as staphopains (SspB and ScpA) are known to inhibit biofilm formation, and the addition of ScpA to bacterial biofilm was shown to be able to disperse an established biofilm^52^. Thus, under conditions with up-regulated staphopain production, these proteases may not only degrade endogenous AMPs, but also counteract biofilm formation and disperse the *S*. *aureus* biofilm. Whether this enables bacterial spread and skin infection under certain circumstances remains to be investigated.

In conclusion, our results demonstrate the existence of *S*. *aureus* biofilms and staphopains in AD skin. The biofilm protects the bacteria from LL-37 mediated killing, and further, staphopains may cleave LL-37 into smaller fragments. Biofilm formation by *S*. *aureus* strains could thus support a persistent bacterial colonization in AD skin. Moreover, *S*. *aureus* biofilms could be a source of proteolytic enzymes at the skin surface, with the capacity to cleave endogenous AMPs and interfering with the epidermal inflammatory response.

## Methods

### LL-37, peptides, SspB and ScpA

LL-37 (LLGDFFRKSKEKIGKEFKRIVQRIKDFLRNLVPRTES) was synthesized by Innovagen AB (Lund, Sweden). The peptides FKR10 (FKRIVQRIKD), FLR11 (FLRN LVPRTES), LLG11 (LLGDFFRKSKE) and FKR21 (FKRIVQRIKDFLRNLVPRTES) were synthetized by Biopeptide (San Diego, CA, USA). The purity of the peptides (>95%) was confirmed by mass spectroscopy analysis (MALDI-TOF Voager). SspB was from Sigma-Aldrich. ScpA was from Preparatis (Krakow, Poland).

### Patients and skin biopsy

Adult patients aged 18 years or over, with AD verified by the UK refinement of the Hanifin and Rajka diagnostic criteria for AD^53, 54^, were recruited from the Dermatology Clinic at Lund University Hospital, Lund, Sweden. Bacterial samples were taken from the skin of the patients and tissue biopsies from AD lesional areas were processed as per standard procedure, for description see section electron microscopy and immuno-staining. The participants gave informed consent complying with the Helsinki Declaration, all methods and experiments were performed in accordance with relevant guidelines and regulations, and the Regional Ethics Examination Board of Lund, Sweden approved the study (Permit Numbers: 144/2010, 317/2010, 82/2012).

### Bacterial isolates

*Staphylococcus aureus* ATCC 29213 isolate was from the American Type Culture Collection (Rockville, MD, USA). Clinical bacterial flora specimens were obtained from the skin of AD patients using a modified version of a method described by Williamson and Kligman^55^. Briefly, a sterile plastic cylinder (3.8 sq cm) was applied to the area of the skin to be scrubbed. Then, a sterile solution of 1 ml 0.05% Triton X-100 in 0.075 M phosphate buffer was pipetted into the plastic cylinder and the area scrubbed for 60 seconds with a sterile Servant^®^ disposable inoculation loop and needle (Konstrumed oy, Tempere, Finland). For identification of bacteria, the samples were processed at the Department of Clinical Microbiology at Skåne University Hospital in Lund, Sweden, following standard routines for identification of *S*. *aureus*. Molecular typing was performed using ADSRRS-fingerprinting analysis as previously described^56, 57^. Briefly, DNA from *S*. *aureus* was digested with the two restriction enzymes BamHI (10 U/μl) (Sigma) and Xbal (10 U/μl) (Sigma). Cohesive ends of DNA were ligated with adapters and then amplified. The PCR products were electrophoresed on polyacrylamide gels and then stained with ethidium bromide. The gels were photographed under UV-light. The strains used in this study represent a variety of different genotypes (Supplementary Table S1).

### Biofilm assay

Quantification of biofilm formation of *S*. *aureus* isolates was performed in a 96-well microtiter plate assay as previously described, with minor modifications^58^ (See Supplementary Methods S1).

### Abiotic solid surface assay (SSA) biofilm formation

To investigate the effect of LL-37 on the biofilm formation of *S*. *aureus* isolates, Abiotic SSA was performed as described by Hell *et al*.^33^ (See Supplementary Methods S1).

### Minimum inhibitory concentration (MIC) and minimal biofilm eradication concentration (MBEC)

Measurement of the bacterial susceptibility was determined by using a modified version of the CBD method (MBEC^TM^ Biofilm Inoculator, Innovotech, Edmonton, Canada)^59^ following the manufacturers protocol. Briefly, to establish biofilms *in vitro* using the CBD, 150 μl bacterial solution of 10^5^ CFU/ml in 1.5% tryptic soy broth (TSB) was supplemented with 0.3% glucose and aliquoted into each well of the MBEC plate and thereafter incubated for 24 h in a rotary incubator at 37 °C and 180 rpm. This allowed the biofilm to form on the pegs. The pegs with established biofilms were then washed once in sterile PBS (200 μl/well). The LL-37 peptide was serially diluted in Müller Hinton broth (MHB) in a 96-well polypropylene microplate (Costar^®^, Corning, NY, USA) and incubated for 16 h according to the NCSLA guidelines, as described by Wiegand *et al*.^60^.

The MIC values measured using the CBD are equivalent to MIC values obtained using the National Committee for Clinical Laboratory Standards (NCCLS) procedure^59^. The MIC value, representing the concentration required to inhibit growth of planktonic bacteria, was determined from bacteria that were shed from the pegs when the lid of the CBD was placed in different concentrations of LL-37. Following determination of the MICs for planktonic cells, the MBEC pegs were washed once in sterile PBS and placed into the recovery plate containing 1.5% TSB supplemented with 0.3% glucose and universal neutralizer following the manufacturers protocol (200 μl/well). The recovery plates were sonicated for 10 min at maximum settings in an ultrasonic bath (Elmasonic S 30 H, Elma Hans Schmidbauer GmbH & Co. KG, Singen, Germany), a new plate cover was then added. MBECs were determined by analyzing the bacterial viability in the biofilm after 24 h of incubation at 37 °C either by reading the turbidity at 600 nm in a 96-well plate reader (Victor^3^ 1420 multilabel counter, Perkin-Elmer) or by obtaining bacterial plate counts. The recorded MIC and MBEC values represent a typical result of three independent experiments.

### Radial diffusion assay (RDA)

RDA was performed as previously described^61, 62^ (See Supplementary Methods S1).

### Fluorescent staining and CLSM

Bacterial cell cultures of *S*. *aureus* isolates were suspended in 3% TSB and shaken at 180 rpm in an incubator at 37 °C for 18 h. A bacterial solution of 10^7^ CFU/ml in 1.5% TSB supplemented with 0.3% glucose was prepared. *In vitro* biofilms were then established by adding 1 ml aliquots of the bacterial solution to sterile Cellview^TM^ cell culture dishes with glass bottom (Greiner Bio-One, Germany), incubated at 37 °C for indicated time periods, and the medium was changed every second day. The medium was removed and the culture dishes were gently washed using PBS solution. For CLMS, the remaining adherent microbial biofilms present on the bottom of the dishes were stained by adding 200 μl/dish of a dilution of 3 μl SYTO^®^ 9 green fluorescent stain (Live/dead^®^ BacLight^TM^ bacterial viability kit L7012, Molecular Probes, USA) into 1 ml distilled water, and incubated at 37 °C for 15 min in the dark. Subsequently, the samples were incubated with 200 μl of 5 μM TAMRA-labeled LL-37 (Innovagen AB, Lund, Sweden), followed by incubation at 37 °C for 15 min in the dark. The samples were then fixed with 2% freshly prepared formaldehyde solution and incubated for 5 min on ice followed by 25 min at room temperature. Subsequently, the material was mounted on glass coverslips using Dako fluorescent mounting medium (S3023, Dako Sweden AB, Stockholm, Sweden). After each step of staining and fixation the biofilm was gently washed twice with PBS. Images were captured using a Zeiss laser-scanning microscope 510 (Carl Zeiss, Jena, Germany). An objective lens (plan aperture, 20x magnification) was used. The image stacks collected by CLSM were analyzed or processed with Zeiss Efficient Navigation (ZEN) 2009 software (Carl Zeiss, Germany) and by the ImageJ software.

### Electron microscopy and immuno-staining

Transmission immunoelectron microscopy was performed as described earlier^63^. In short, specimens were fixed in 150 mM sodium cacodylate, 2.5% glutaraldehyde, pH 7.4 and embedded in Epon. After antigen retrieval with sodium metaperiodate, specimens were incubated with primary antibodies, followed by detection with species-specific secondary antibody-gold conjugates. Samples were examined in a Philips/FEI CM 100 TWIN transmission electron microscope (FEI Co, Hillsboro, Oregon, USA) at 60-kV accelerating voltage. Images were recorded with a side-mounted Olympus Veleta camera with a resolution of 2048 × 2048 pixels (2 k × 2 K) using ITEM^TM^ software. For scanning electron microscopy, specimens were fixed over night at RT with 2.5% glutaraldehyde in 150 mM cacodylate, pH 7.4. After washing with cacodylate buffer, they were dehydrated with an ascending ethanol series from 50% (v/v) to absolute ethanol, and subjected to critical point drying with carbon dioxide. Tissue samples were mounted on aluminum holders, sputtered with 20 nm palladium/gold, and examined in a Philips/FEI XL 30 FESEM scanning electron microscope using an Everhart-Tornley secondary electron detector or in DELPHI, Phenom-World. Image processing was done with the Scandium software for simple image acquiring and auto-storage into the Scandium database. All electron microscopic work was performed at the Core Facility for Integrated Microscopy, Panum Institute, University of Copenhagen and at Infection Medicine, Lund University. Contrast, brightness and pseudocolours were adjusted in Adobe Photoshop CS6.

### Anti-ScpA and anti-SspB monoclonal antibodies

ScpA and SspB were isolated from the culture medium of the V8 strain of *S*. *aureus* or isogenic double *sarA* and *scpA* mutant of *S*. *aureus* 8325–4 deficient in ScpA and overexpressing SspB as described previously^64, 65^. Alternatively, prostaphopain B was cloned into pGEX-5T, expressed, processed by V8 protease and purified as described by Filipek *et al*.^66^. Anti-ScpA (IgG_1_, 1A3) and anti-SspB (5E12 and 13G12, all IgG_1_) monoclonal antibody (MAb) were produced in a monoclonal facility at the University of Georgia (Athens, GA).

***Ex vivo*** **model, quantification of strongly adherent bacteria in lesional AD skin** (See Supplementary Methods S1).

**NF-κB/AP-1 activation assay and MTT assay** (See Supplementary Methods S1).

**Proteolytic degradation of LL-37 and SDS-PAGE analysis** (See Supplementary Methods S1).

### Mass spectrometric identification of LL-37-derived peptides

Matrix-assisted laser desorption ionization mass spectrometry (MALDI-MS) was used for the identification of trypsin generated peptides of LL-37. A MALDI matrix solution consisting of 5 mg/ml hydroxycinnamic acid in 50% acetonitrile, 0.1% (v/v) phosphoric acid^67^ was mixed with the staphopain-generated peptides. The matrix solution contained two peptide standards [des-arg-bradykinin (m/z 904.468) and ACTH 18–39 (m/z 2465.199)] that were used for internal mass calibration in every analyte/matrix position. MALDI-MS and MS/MS analyses of the samples were performed on a 4700 Proteomics Analyzer MALDI-TOF/TOF™ mass spectrometer (Applied Biosystems, Framingham, MA). Database searching was carried out using Mascot (Matrix Science) with Swissprot as the database and with a peptide mass tolerance of 50 ppm and a fragment mass tolerance of 0.2 Da.

### Statistics

Data are presented as means ± standard deviation of the means. To describe the differences between groups, one-way ANOVA with Dunnet’s multiple comparisons test was used. In order to determine significant differences between two groups, the Wilcoxon rank-sum test was used, and p < 0.05 was considered as significant. The statistical software used was GraphPad PRISM^®^ version 6.0c (GraphPad Software, Inc., La Jolla, CA, USA).

### Data Availability

The datasets generated during and/or analyzed during the current study are available from the corresponding author on reasonable request.

## Electronic supplementary material

Supplementary Information
