# Supplementary material for: Identification of bacterial biofilm and the Staphylococcus aureus derived protease, staphopain, on the skin surface of patients with atopic dermatitis
Source: Sci Rep. 2017 Aug 18;7:8689. doi: 10.1038/s41598-017-08046-2 (PMC5562790; doi:10.1038/s41598-017-08046-2)
Supplement: Supplementary file 1 — Supplementary Information [file 41598_2017_8046_MOESM1_ESM.pdf]

Title: Identification of bacterial biofilm and the *Staphylococcus aureus* derived protease, staphopain, on the skin surface of patients with atopic dermatitis.

Authors: Andreas Sonesson, Kornelia Przybyszewska, Sigrid Eriksson, Matthias Mörgelin, Sven Kjellström, Julia Davies, Jan Potempa, Artur Schmidtchen

Supplementary information:

Supplementary Methods S1

Bacterial growth of the S. aureus isolates

One hundred µL of a bacterial solution containing 10^9 CFU/ml of *S. aureus* were transferred to 10 ml of fresh 3% TSB and incubated at 37°C. For monitoring bacterial growth, the optical density of the liquid culture was measured spectrophotometrically (600 nm) at different time points.

Biofilm assay

Overnight cultures of *S. aureus* were diluted 1:1000 in 1.5% tryptic soy broth (TSB) (Difco, Detroit, MI, USA) supplemented with 0.3% (w/v) glucose. Five µl aliquots of the bacterial suspension were added to 100 µl medium, 1.5% TSB supplemented with 0.3% (w/v) glucose, in each well of a 96-well polyvinyl chloride plate with round bottom wells (Costar, Corning, USA). Negative control wells contained medium only. The plate was incubated at 37 °C for 24 h, and the planktonic cells were removed before the washing the wells gently with tap water. The biofilms were stained with crystal violet (1% w/v in H_2O, Sigma, St. Louis, MO, USA) for 15 min. The crystal violet solution was removed and the plate washed with tap water. Subsequently, the biofilms were incubated in absolute ethanol for 15 min to extract the dye. To quantify the amount of biofilm, this solution was transferred to a new flat-bottomed plate and the absorbance was measured at 600 nm in a Victor3 1420 multilabel counter (Perkin-Elmer, USA). The different bacterial isolates were analyzed in duplicate at least three times. The isolates were classified as weak biofilm producers if they gave an OD_{600} value below 0.4 or as good biofilm producers if presented an OD_{600} value of more than 0.4.

Abiotic solid surface assay (SSA) biofilm formation

Biofilm formation was measured in 96-well polystyrene microtitre plates with round bottom wells (BectonDickinson, Franklin Lakes, NJ, USA). One hundred µL of a *S. aureus* solution containing a total of 5 x10^4 cells was added to each well, together

with different concentrations of LL-37 (in 100 μL, 0.05% acetic acid). The growth media was composed of 1.5% TSB supplemented with 0.3% (w/v) glucose and 0.05% acetic acid in a total volume of 200 μl. After incubation for 20 h at 37 °C, the 96-well microtitre plates with biofilm were washed twice with phosphate buffered saline (PBS), to remove the media and the planktonic cells. The biofilms were stained with crystal violet (0.1% w/v in dH_2O, Sigma, St. Louis, MO, USA) for 15 min. The plates were then washed in PBS twice and subsequently incubated in 95% ethanol for 15 min to solubilize the dye. To quantify the amount of biofilm, the absorbance was measured at 600 nm. The different bacterial isolates were tested in duplicates at least twice.

Radial diffusion assay

The underlay gel, 0.03% (w/v) TSB, 1% (w/v) low-electroendosmosistype (Low-EEO) agarose (Sigma, St Louis MO), 0.02% Tween-20 was poured into a ∅ 85 mm petri dish**.** After the agarose solidified, 4 mm-diameter wells were punched and filled with 6 μl of test sample or buffer only. Plates were then incubated at 37 °C for 3 h to allow diffusion of the peptides. The underlay gel was then covered with 5 ml of molten overlay gel (6% TSB and 1% Low-EEO agarose in dH_2O). Antimicrobial activity of a peptide was visualized as a clear zone around each well after 18-24 h of incubation at 37°C.

Ex vivo model, quantification of strongly adherent bacteria in lesional AD skin.

Punch biopsies (4mm) derived from lesional skin of patient with AD, and with verified colonization of the lesions by *S. aureus*, were used for quantification of weakly and strongly attached skin associated bacteria. The biopsies were divided in 4 equal pieces and incubated in sterile filtered tap water 37 °C for 1 h, then removed from the water and washed in 100 μL PBS. Subsequently the biopsies were sonicated for 3 min at maximum settings in an ultrasonic bath (Elmasonic S 30H. Elma Hans Schmidbauer GmbH & Co. KG, Singen, Germany) and vortexed for 60 s. The supernatants were plated on Todd-Hewitt (Becton, Dikinson, Sparks, MD, USA) agar plates and CFU were enumerated after overnight growth. The results obtained are reported as CFU per square centimetre of skin.

Proteolytic degradation of LL-37 and SDS-PAGE analysis

LL-37 (3 μg), was incubated with *S. aureus* staphopain B (0.6 μg) (Sigma-Aldrich, St. Louis, MO, USA) in a total volume of 30 μl for 3 h at 37 °C. LL-37 alone was used as a control. The materials were subsequently analysed on Novex®10-20% Tricine Gel (Life technologies, Carlsbad, CA, USA) under reducing conditions.

NF-κB/AP-1 activation assay

The NF-κB/AP-1 reporter cell line THP1-XBlue™-CD14, derived from the human monocytic THP-1 cell line, (InvivoGen, San Diego, CA, USA) was cultured according to the manufacturer's protocol. The cells were stimulated with 100 ng/m *E. coli* LPS (0111:B4) (Sigma-Aldrich, approximate 500.000 endotoxin units/mg) or 1μg/ml *S. aureus* LTA (# tlrl-pslta) (Invivogen, San Diego, CA, USA) together with the indicated concentrations of FKR21 for 20-24 h. NF-κB/AP-1 activation was assessed by using QUANTI-Blue™ assay according to the instructions supplied by the manufacturer. Secreted embryonic alkaline phosphatase (SEAP) was produced by the cell line upon stimulation and assessed by a SEAP detection reagent in cell supernatants. Absorbance at 600 nm was then detected.

MTT assay

For analysing cell viability after treatment with the LL-37 derived peptide fragments the MTT assay was used. Sterile filtered MTT (3-(4,5-dimethylthiazolyl)-2,5-diphenyl-tetrazolium bromide; Sigma–Aldrich, St.-Louis, USA) solution (5 mg/mL in PBS) was stored protected from light at −20 °C until use. HaCaT keratinocytes were grown in 96-well plates at a density of 20 000 cells/well in Keratinocyte Cell Basal Medium (KBM-Gold) with supplements and growth factors (#00192152, Lonza Walkersville, MD, USA) for 22 h in CO_2 at 37 °C. After 24 h incubation with the LL-37 derived peptide fragments (2-20 μM), 10 μL MTT was added to each well and incubated for 2 h in CO_2 at 37 °C. The MTT containing medium was then removed. The blue formazan product generated was dissolved through addition of 100 μL of 100% dimethylsulfoxide (Duchefa, Haarlem, The Netherlands) per well. The plates where always protected from light and gently swirled for 30 min in RT to resolve the precipitate. The absorbance was monitored at 550 nm and the results represent mean values of three independent experiments.

Supplementary Table S1. The biofilm production and genotypes of the skin derived *S. aureus* isolates from patients with AD used in the study.

Biofilm-production OD_{600nm} Mean (SD)	Genotype ADSRRS finger-printing	*S. aureus* isolate ID
0.12 (0.01)	I	19
0.12 (0.02)	L3	30
0.14 (0.03)	M	31
0.16 (0.04)	N	5
0.17 (0.04)	S	4
0.20 (0.06)	S	3
0.20 (0.06)	T	7
0.20 (0.02)	A	11
0.21 (0.06)	S	2
0.21 (0.05)	G2	32
0.22 (0.04)	L1	26
0.24 (0.05)	H1	25
0.25 (0.09)	B	20
0.27 (0.06)	G1	1
0.27 (0.06)	W	6
0.29 (0.07)	K	14
0.32 (0.10)	G	8
0.33 (0.08)	C	18
0.35 (0.05)	P	27
0.36 (0.10)	A	15
0.41 (0.08)	H	17
0.42 (0.08)	G	16
0.47 (0.09)	T	12
0.53 (0.11)	L2	29
0.56 (0.14)	W	28
0.57 (0.14)	P1	23
0.62 (0.18)	U	22
0.63 (0.11)	H	9
0.76 (0.20)	E	10
0.89 (0.09)	E	13
1.08 (0.30)	S	21
1.08 (0.11)	L	24

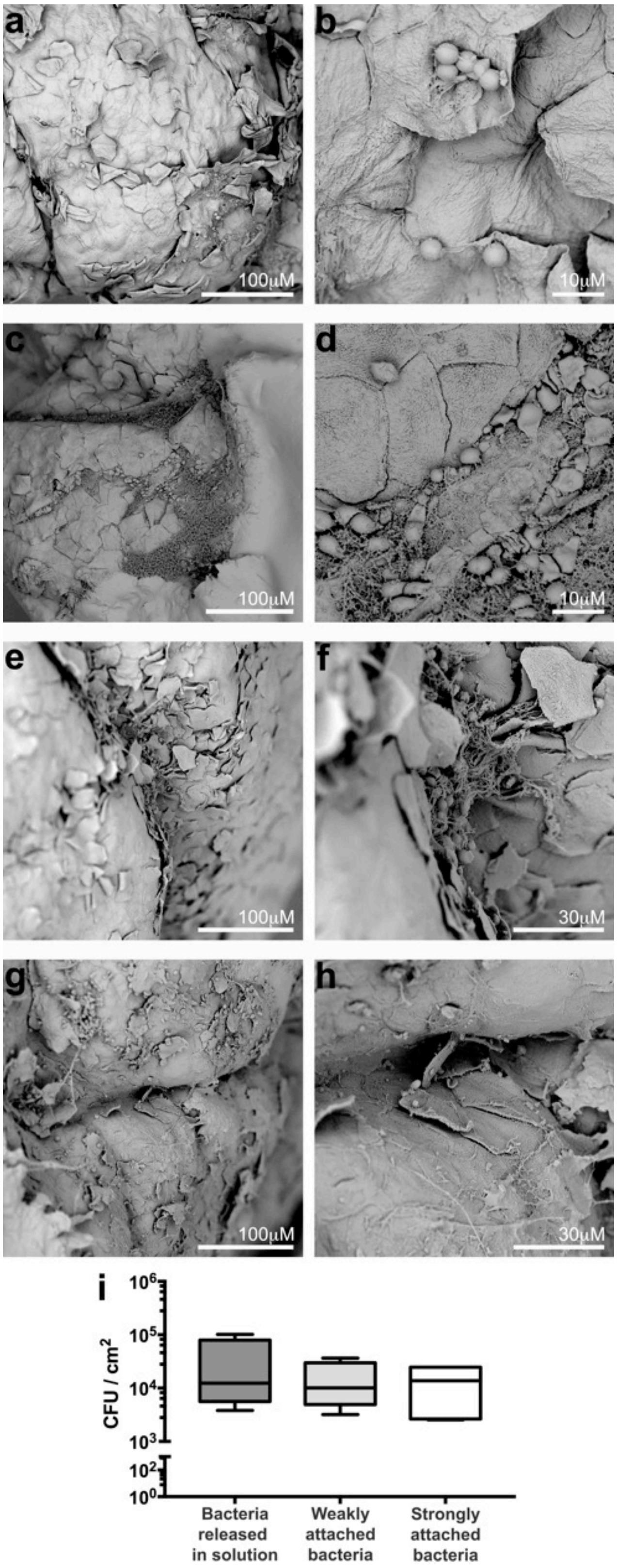

Supplementary Fig. S1. SEM of skin biopsies derived from non-AD and AD patients, and *ex vivo* quantification of skin associated bacteria derived from lesional AD skin. (a) Skin biopsy derived from non-lesion skin of one AD patient and (b) the corresponding high magnification picture, visualizing stratum corneum with minor extracellular material and insignificant bacterial colonization. (c, e, g) Skin biopsies derived from lesional AD skin of three patients colonized with *S. aureus* and

(d, f, h) the corresponding high magnification pictures showing stratum corneum with biofilm composed of intricate extracellular matrix material, fibrin and bacteria observed by SEM. (i) Total bacterial count of bacteria released from the biopsies when incubated in sterile filtered tap water for 1 h (dark grey box). Subsequently the biopsies were washed with PBS (light grey box), and then sonicated and vortexed (white box), the supernatants were plated on agar plates and CFU were enumerated after overnight growth, results obtained are reported as CFU per square centimetre of skin, *n*=4.

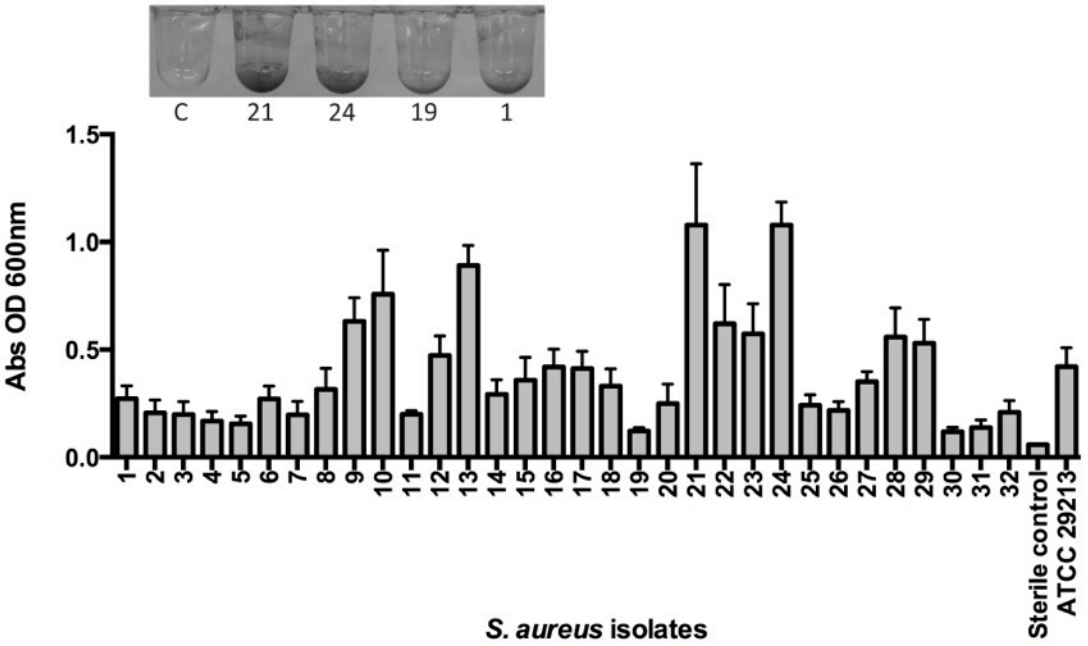

Supplementary Fig. S2. Biofilm formation by isolates derived from patients with AD. Biofilm formation after 24 h of incubation of the *S. aureus* isolates were measured using the crystal violet assay and expressed as optical density (OD 600nm). The 32 clinical *S. aureus* isolates derived from skin of AD patients showed different ability to form biofilm *in vitro*. The *S. aureus* strain ATCC29213 was included as a reference. Mean values for the biofilm formation in three independent experiments, along with standard deviations, are presented. The inset illustrates one column of a 96-well plate with examined *S. aureus* isolates after crystal violet staining of the biofilm adhering to the plastic in the wells. From left, C, sterile control, 21 and 24, two biofilm producing *S. aureus* isolates derived from skin of AD patients, and 19 and 1 two low biofilm producing isolates.

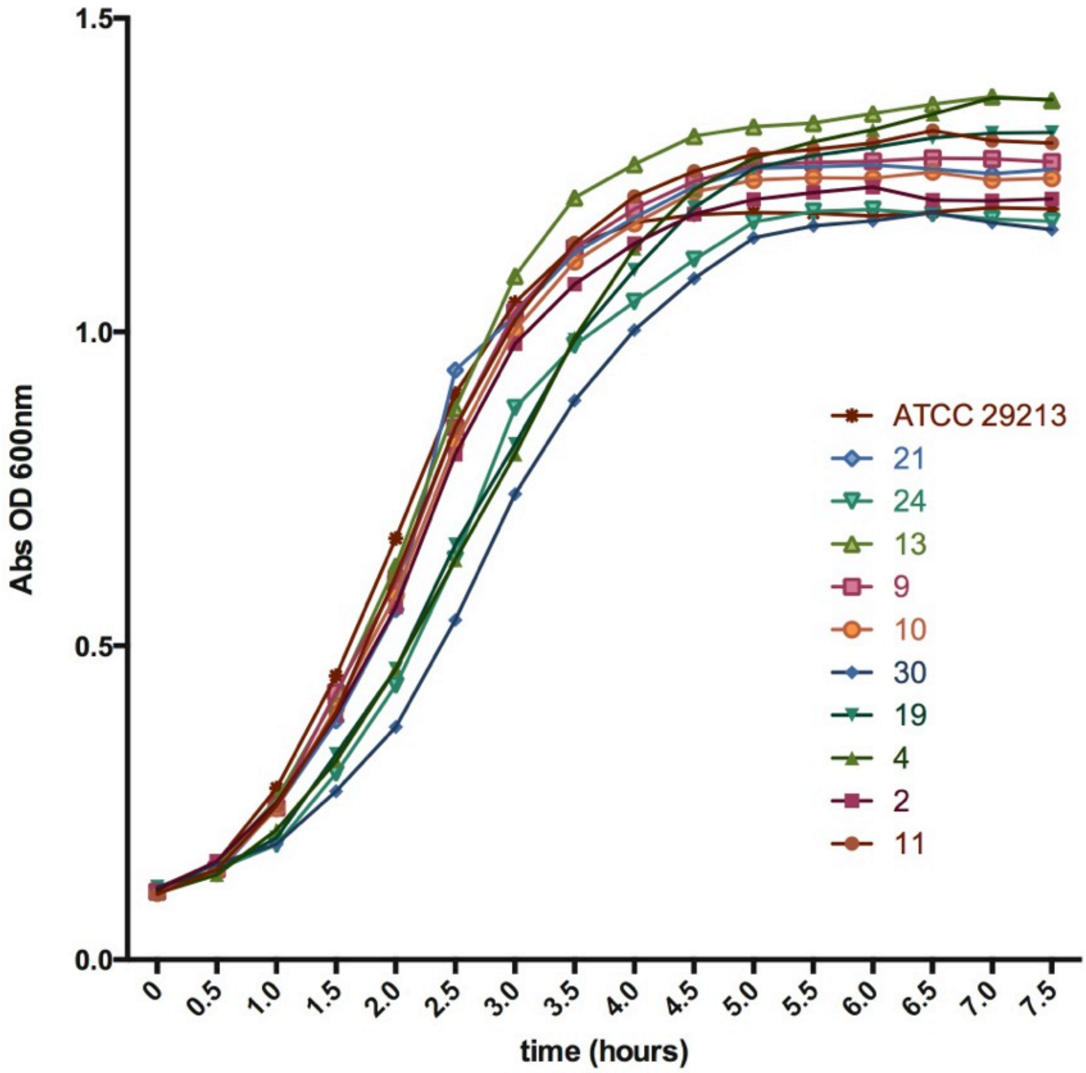

Supplementary Fig. S3. Bacterial Growth. Bacterial growth expressed as optical density (OD 600 nm). The growth of five low and five high biofilm producing *S. aureus* isolates from patients with AD, as well as the ATCC29213 *S. aureus* strain, are visualized. Results are representative of three independent experiments.

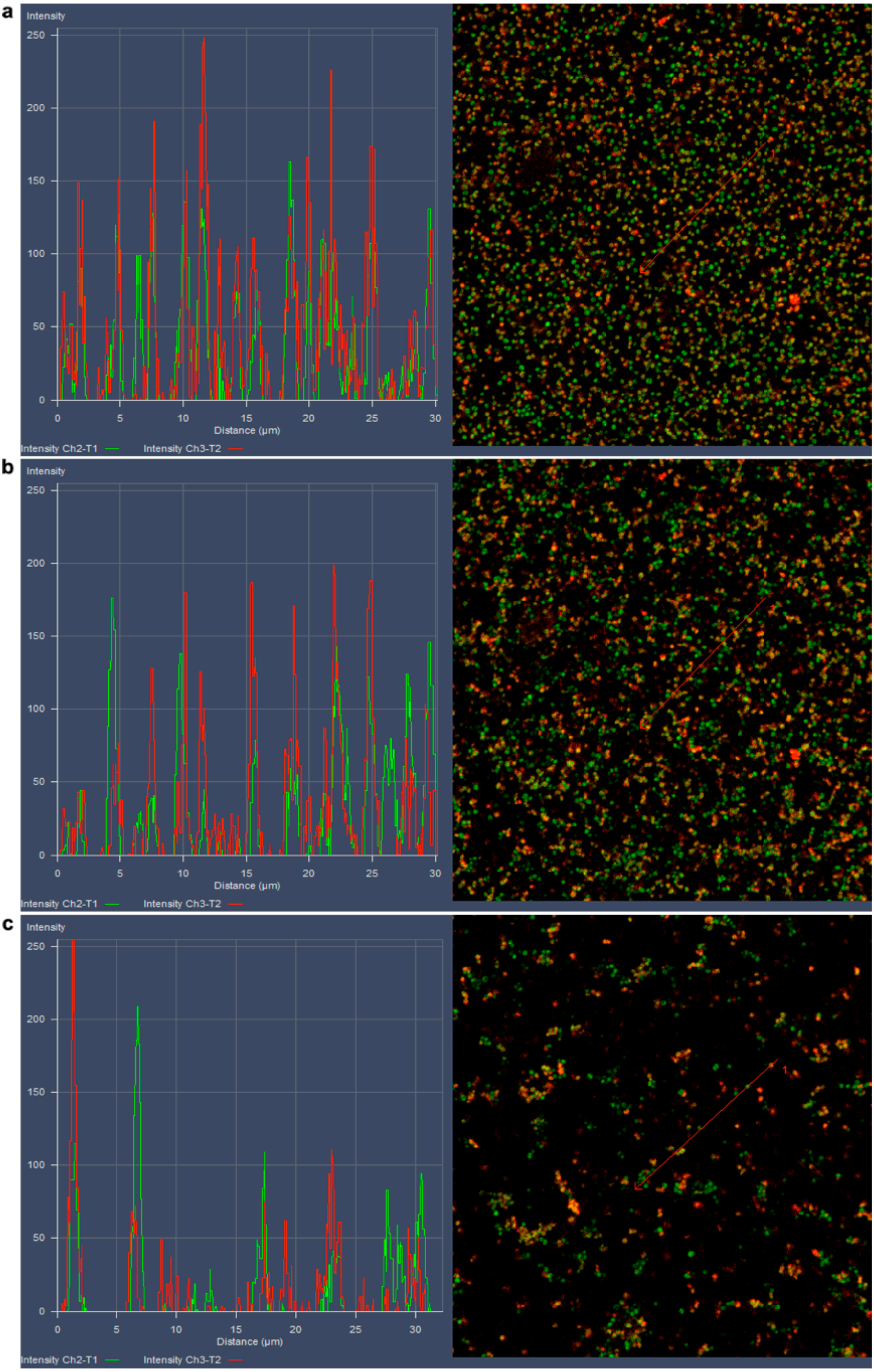

Supplementary Fig. S4. TAMRA labeled LL-37 binding to *S. aureus* cells throughout the biofilm. Z-stacks were analyzed, and quantification of the intensity using the *Profile* plug-in (Zeiss ZEN Confocal Software) was done. Results indicated three distinctly different populations of fluorophores in the samples; red (TAMRA-

labeled LL-37) and green (*S. aureus* cells) seen separately, and yellow representing co-existence of bacterial cells and the TAMRA-labeled LL-37 peptide. Binding of TAMRA labeled LL-37 to the *S. aureus* cells were present throughout the biofilm. Three examples (a-c) collected at different depths along the z-axis, derived from the biofilm presented in figure 2b, are shown.

a

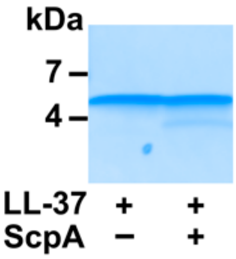

b

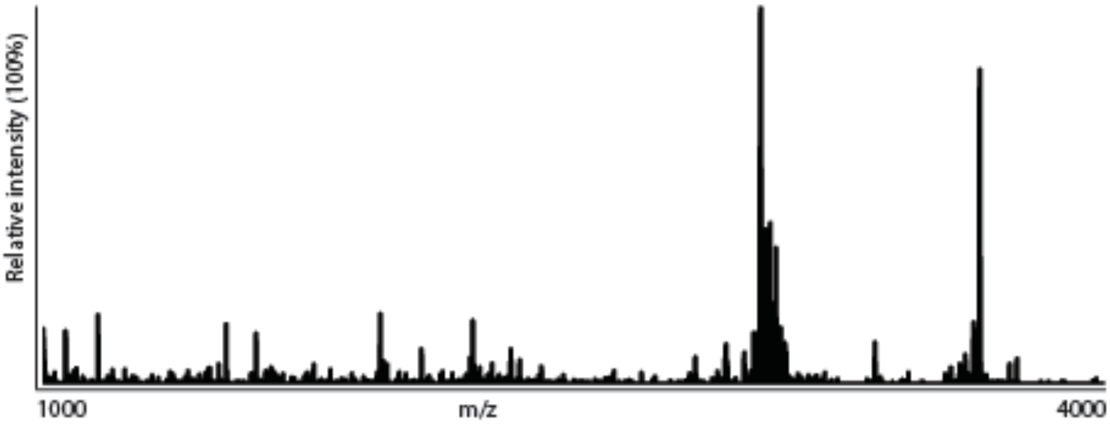

Supplementary Fig. S5. Degradation of LL-37 by staphopain A (ScpA). LL-37 was incubated with and without *S. aureus* ScpA and analyzed using SDS-PAGE (Novex®10-20% Tricine Gel) (a), and by mass spectrometry (b).

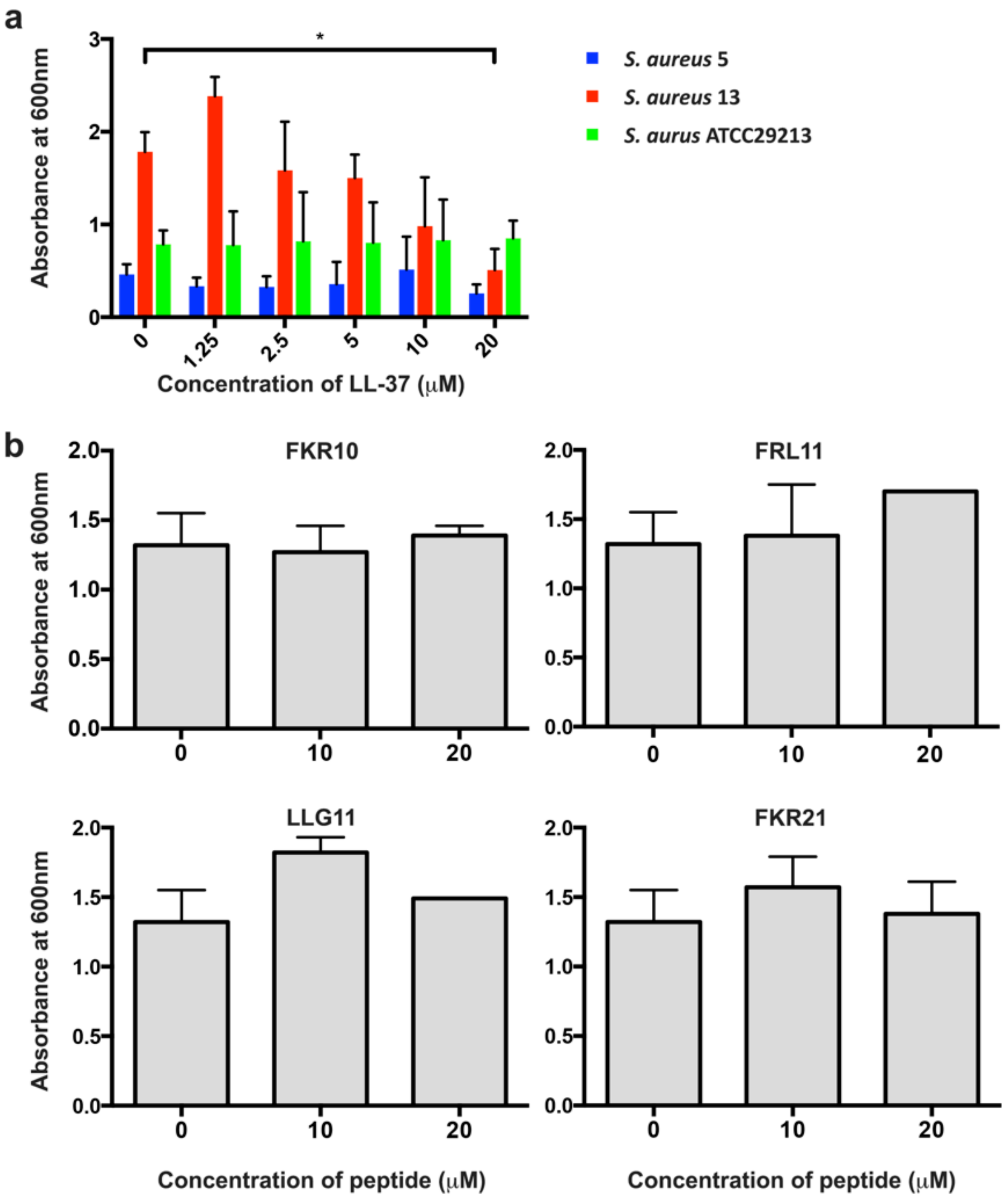

Supplementary Fig. S6. LL-37 and effects on biofilm production.

(a) Analysis of biofilm formation in the presence and absence of LL-37. High (*S. aureus* 13) and low (S. *aureus* 5) biofilm producing clinical isolates of *S. aureus* as well as the ATCC strain 29213 are subjected to different concentrations of peptide in an abiotic SSA, error bars represent the mean ± SD. (b) Analysis of biofilm formation in the presence and absence of the peptides FKR10, FLR11, LLG11 and FKR21. A high biofilm producing clinical isolate of *S. aureus* (*S. aureus* 13) is subjected to different concentrations of peptide in an abiotic SSA, error bars represent the mean ± SD. * $P<0.05$.

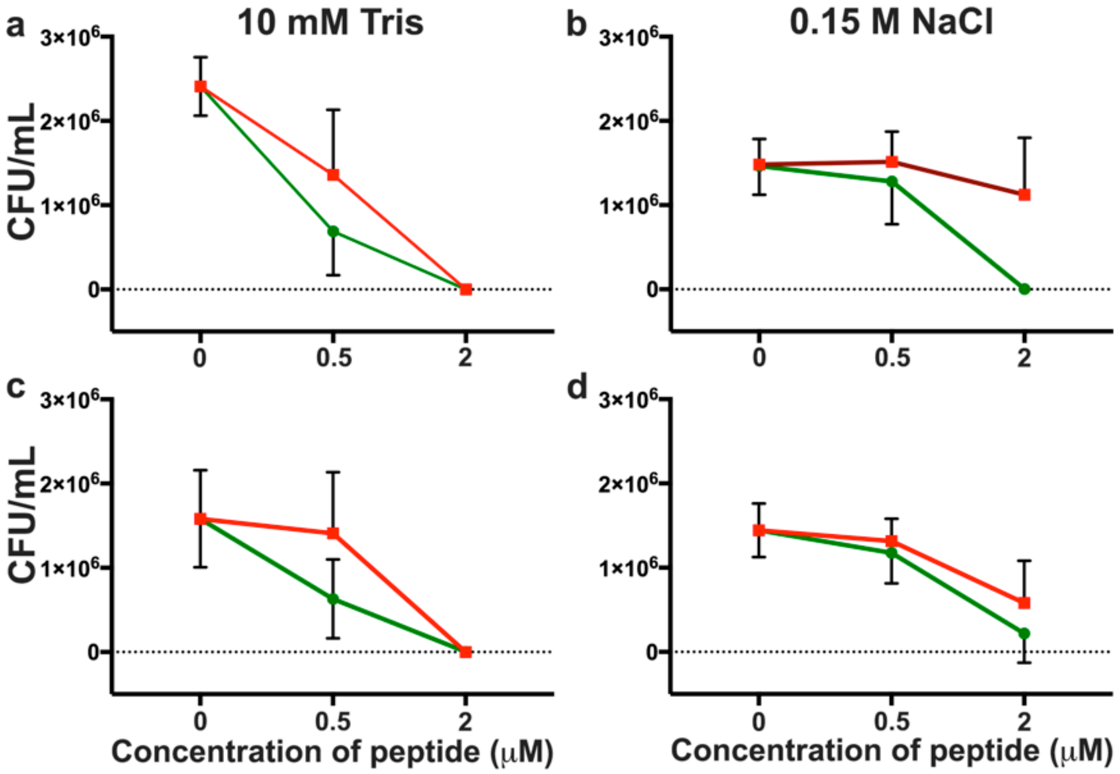

Supplementary Fig. S7. Effects of LL-37 and FKR21 in low and physiologic salt conditions. *S. aureus* ATCC 292913 (a, b) and *S. aureus* clinical isolate 13 (c, d) subjected to LL37 (green lines) and FKR21 (red lines) in low salt (10 mM Tris buffer) and physiologic salt conditions (0.15 M NaCl), *n*=3.
